# Association between Combination Antiretroviral Therapy and Telomere Length in People Living with Human Immunodeficiency Virus

**DOI:** 10.3390/biology12091210

**Published:** 2023-09-05

**Authors:** Ena Bukic, Jelena Milasin, Bosko Toljic, Jelena Jadzic, Djordje Jevtovic, Bozana Obradovic, Gordana Dragovic

**Affiliations:** 1 Department of Pharmacology, Clinical Pharmacology and Toxicology, University of Belgrade Faculty of Medicine, 11000 Belgrade, Serbia; 2 Department of Human Genetics, School of Dental Medicine, University of Belgrade, 11000 Belgrade, Serbia; 3 Center of Bone Biology, University of Belgrade Faculty of Medicine, 11000 Belgrade, Serbia; 4 Infective and Tropical Diseases Hospital, University of Belgrade Faculty of Medicine, 11000 Belgrade, Serbia

**Keywords:** HIV, antiretroviral therapy, cART, telomere, telomere length, RTL

## Abstract

**Simple Summary:**

Inevitable aging is accompanied by progressive decline in vital functions. People living with HIV infection may experience accelerated aging caused by constant state of inflammation and long-term use of medications that keep HIV infection under control. Human chromosomes end with telomeres—structures that shorten at a steady rate during life. Various harmful influences can contribute to telomere shortening. When telomere length reaches its minimum, the cell dies. If large numbers of cells die, the organism will age rapidly. By monitoring the length of telomeres, it is possible to estimate whether any harmful effect leads to premature aging, decline in the body’s vital functions, and the occurrence of diseases associated with aging. The goal of our study was to identify which medication, used to treat HIV infection, together with changes in the organism, caused by HIV infection, could lead to eventual changes in telomere length. The results of this research could potentially indicate the need to avoid the prescription of efavirenz, which in our study was associated with shorter telomeres, so shortening can be prevented in patients who have never used efavirenz or stopped in patients who already use it.

**Abstract:**

Long-term exposure to combination antiretroviral therapy (cART) may be associated with accelerated ageing. Telomere length is considered to be reliable aging biomarker. The aim of this study was to compare patients’ relative telomere length (RTL) between and within different cART classes and to estimate the impact of certain HIV-related variables on RTL. The study was conducted in 176 HIV-infected male patients receiving cART, with ≤50 copies HIV RNA/mL plasma. RTL was determined from mononuclear cells by quantitative polymerase chain reaction. Standard statistical tests and unsupervised machine learning were performed. The mean RTL was 2.50 ± 1.87. There was no difference (*p* = 0.761) in RTL between therapeutic groups: two nucleoside reverse transcriptase inhibitors as the backbone treatment, combined with either integrase inhibitor, protease inhibitor, or non-nucleoside reverse transcriptase inhibitor (NNRTI). Machine learning results suggested duration of HIV infection, CD4+ T-cell count, and cART, including NNRTI, as potentially significant variables impacting RTL. Kendall’s correlation test excluded duration of HIV infection (*p* = 0.220) and CD4+ T-cell count (*p* = 0.536) as significant. The Mann–Whitney test confirmed that cART containing NNRTI impacted RTL (*p* = 0.018). This was the first study to show that patients using efavirenz within cART had significantly shorter telomeres than patients using nevirapine.

## 1. Introduction

According to the World Health Organization (WHO), there were 38.4 million people living with human immunodeficiency virus (HIV) at the end of 2021 [1]. The introduction of antiretroviral therapy has led to substantial improvements in the life expectancy of people living with HIV, which is now treated as a chronic disease requiring life-long antiretroviral treatment [2,3,4]. Life expectancy at age 20 years of HIV-infected individuals has increased from 11.8 years in the monotherapy era to 54.9 years in the most recent combination antiretroviral therapy (cART) era, reaching up to 91.5% of the matched general population life expectancy [5]. cART suppresses viral replication and lowers the HIV plasma viral load in blood by targeting several key stages of the virus life cycle. Both gold standard and most recent international guidelines suggest that the first line cART includes two drugs from the group of nucleoside reverse transcriptase inhibitors (NRTI) and one integrase inhibitor (INSTI); or two NRTIs and one protease inhibitor (PI); or two NRTIs and one non-nucleoside reverse transcriptase inhibitor (NNRTI) [6,7]. 

Since antiretroviral drugs cannot be successfully transported through all tissue barriers, local reservoirs are formed in the body in which the virus continues to replicate, leading to a permanent HIV presence in the body [8]. Consequently, the persistent presence of HIV in the body causes chronic inflammation and continuous activation of the immune system [9]. In long-term exposure to cART, side effects have cumulative toxic effect that burden the organism. This can stimulate progressive pathophysiological mechanisms which are potentially related to the accelerated aging processes. It is known that the biological age of HIV-infected patients differs from their chronological age [10] and that untreated HIV infection can induce biological age advancement of up to 14.7 years [11]. The difference between the biological and chronological age of patients can be monitored using cell aging markers, namely telomere length [12]. Telomeres are physiologically shortened with each cell division. When the critical shortening is reached, divisions are no longer possible, and the cell undergoes apoptosis [13]. The length of telomeres under physiological conditions is maintained by the enzyme telomerase [14]. The effect of physiological, psychological and pathological stressors, antiretroviral drugs, as well as chronic inflammation contributes to telomere shortening [15,16].

So far, it has been confirmed that short telomere length in HIV-infected patients is associated with persistent immune activation [17] and lower immunological response, sometimes despite the suppressive cART [18]. The molecular mechanisms through which antiretroviral drugs can affect telomere shortening are diverse. For example, efavirenz contributes to telomere shortening by disrupting cellular homeostasis [19], stimulating the production of reactive oxygen species [20], increasing inflammation [21] and inhibiting the molecules that stabilize telomerase [22]. Unlike efavirenz, tenofovir directly inhibits telomerase [23], but also changes the expression of genes involved in telomere maintenance [24]. Dolutegravir, on the other hand, can indirectly shorten telomere by negatively regulating telomerase activity and promoting the production of reactive oxygen species [25]. However, a few studies have shown that cART is beneficial for telomere length recovery [26,27].

Untreated HIV infection and ageing had a synergistic effect on telomere shortening. It was calculated that a viral load of >100,000 copies/mL of blood plasma affected telomere length similarly to 7 years of ageing and concluded that an HIV-infected person with a viral load >100,000 copies/mL compared to an HIV-negative person with the same chronological age could have the telomere length of a person almost two decades older [28]. Untreated HIV infection will progress. To understand the progression of HIV infection, the number of CD4+ T cells is monitored [29]. The number of CD4+ T cells < 200 cells/µL was associated with shortened telomeres [28]. The parameter often used to track inflammation [30] and the progression of HIV infection [29] is C-reactive protein (CRP). The influence of CRP on telomere length is not yet clear, as there are studies showing shortening [31], but also showing no influence of CRP on telomere length [32]. In addition to cART, due to their susceptibility to the development of various comorbidities, HIV-infected patients also use other medicines to keep associated diseases under control. It has been shown that some medicines can affect telomere length. Although statins, providing efficacy in the setting of cardiovascular diseases and atherosclerosis, which represent very common HIV-related comorbidities, have been reported to lead to telomere shortening [28], the mechanisms through which they can preserve telomere length are also suggested [33]. As well as statins, the influence of insulin on the length of telomeres cannot be generalized because there is evidence that it can lead to telomere shortening [34], but also that it can have a protective effect on telomeres [35]; while diabetes is undoubtedly associated with telomere shortening [36]. Diabetes is often associated with obesity. The influence of obesity on telomere length should not be neglected either. In a meta-analysis that included almost 150,000 subjects, not only was the inverse relationship between BMI and telomere length shown, but it was also calculated that with each unit increase in BMI, telomere length shortens by 3.99 bp [37].

The aim of this study was to compare relative telomere length (RTL) between patients taking different antiretroviral regimens within cART and to determine which HIV-related variable with potential influence on telomere length and aging affects telomere length the most.

## 2. Materials and Methods

This cross-sectional study was conducted on 176 HIV patients, ≥18-years old Caucasians, all males, regularly monitored and treated at the Center for HIV/AIDS of the Kosta Todorovic Infectious and Tropical Diseases Hospital, University Clinical Center of Serbia. The study was approved by the ethics committee of the Faculty of Medicine, University of Belgrade (1322/IX-24).

HIV infection was confirmed with real-time polymerase chain reaction (PCR). Patients had been receiving cART for at least 6 months, and all had ≤50 copies of HIV RNA/mL plasma. The presence of an acute infection, hepatitis B virus infection (HBV), hepatitis C virus infection (HCV), human papillomavirus (HPV) infection, or radiotherapy application, cytotoxic drug usage, recreational drug use, and chronic alcoholism were exclusion criteria. Clinical data from patient records and venous blood samples were collected after patients had signed the written informed consent for participation and confirmed that they understood its content.

Using standard venipuncture methods, blood samples (4 mL) were collected from each patient in K2EDTA and serum tubes and inactivated according to a standard protocol [38]. Mononuclear cells were extracted using a hydrophilic polysaccharide, according to the manufacturer’s recommendations (Saint Louis, MO, USA) [39]. In short, mononuclear cells were separated from plasma, erythrocytes, and granulocytes blood using a hydrophilic polysaccharide on a density gradient. Each blood sample was first diluted with saline solution (ratio 1:1). Polysaccharide separation reagent was added to the diluted blood and subjected to centrifugation (400× *g*, 30 min). After centrifugation, the mononuclear cells were extracted into new tubes.

DNA molecules were isolated from mononuclear cells by the salting out method [40], after both the concentration and quality of isolated DNA were evaluated by the spectrophotometric method (BioSpec-nano, Shimadzu Biotech, Kyoto, Japan). Only samples with sufficient DNA concentration and satisfactory purity were used in further analysis [41].

RTL was determined by the quantitative PCR method (qPCR), relative to the reference unique sequence—human beta-globin (HBG). qPCR was performed in a single reaction for telomeres and HBG, using the Line GeneK Fluorescence Real-time PCR Detection System (BIOER Technology, Shanghai, China) and reagents shown in Table 1.

Briefly, a modified Cawthon model was used. The method involved in vitro multiplication of selected sequences, in this case telomeres and the HBG gene, through 30 cycles (30 in our case) in which the phases of denaturation (95 °C, 5 min), hybridization (95 °C, 15 s), and elongation (60 °C, 1 min) of the DNA sequences alternate. Two master mixes of PCR reagents were prepared, one with the forward and reverse telomere primer pair, the other with the forward and reverse HBG primer pair. Thermal cycler that performs the replication reaction detects the fluorescence produced by the incorporation of a fluorescent dye into the DNA sequence during each repeated cycle of replication. The cycle in which the multiplication reaction enters the exponential phase represents the Ct value (Cycle threshold). The Ct value is inversely proportional to the initial amount of DNA material being amplified, so a lower Ct value will indicate a higher initial concentration of the DNA sequence being amplified, and a higher Ct value will indicate a lower initial concentration of the DNA sequence. In each qPCR reaction, reference tumor DNA (293T) was used in serial dilution, 2.5 ng/μL, 5 ng/μL, 10 ng/μL, 20 ng/μL, 40 ng/μL, in order to obtain a standard curve and monitor reaction efficiency. Only standard curves with a slope from −3.2 to −3.7 and a linear correlation coefficient (R2) ≥ 0.98 were accepted. Ct values (mean value) obtained for telomeres and HBG were used to calculate the T/S ratio (number of telomere repeats/number of genes present in one copy, HBG). The relative T/S ratio is obtained using the T/S = 2^−ΔΔCt^ formula, where ΔΔCt = (Cttelomeres − CtHBG) sample − (Cttelomeres − CtHBG) reference DNA (10 ng/μL dilution used for the standard curve). This way, RTL was obtained for each sample [42]. The sequences, concentrations and annealing temperature of the primers are shown in Table 2.

The cut-off values used to determine the status of hyperlipidemia were as follows: cholesterol level >5.2 mmol/L and triglyceride level >1.7 mmol/L [43]. Glycemia values above 6.1 mmol/L were considered elevated [44]. Arterial hypertension is characterized by a pressure value of ≥140 mmHg [45]. Cut-off values of the mentioned markers are determined by national guidelines issued by the Ministry of Health of the Republic of Serbia.

Data obtained by clinical examination and laboratory tests were processed and visualized in R programming language for statistical computing (R Core Team, Vienna, Austria). Data were subjected to unsupervised machine learning, which uses algorithms to analyze, cluster, and discover patterns or data groupings without the need for human intervention. For machine learning, the ‘randomForest’ [46] and ‘Boruta’ [47] packages were used. For the statistical testing of telomerase length, differences between groups, non-parametric Kruskal–Wallis (for three or more groups) and Mann–Whitney (for two groups) tests were performed using built-in R package ‘stats’ version 3.6.2 (R Core Team, Vienna, Austria), followed by Dunn’s post-hoc test with Holm’s correction for multiple comparisons [48]. Correlation between two quantitative variables was tested using Kendall’s test, while visualization of statistical testing results was performed using the ‘ggrpub’ R package [49]. For all statistical tests, the significance level was set at 0.05 and adjusted in the case of multiple comparisons.

## 3. Results

The patients’ mean age was 42.70 ± 13.18 years and their mean body mass index (BMI) was 24.51 ± 3.49 kg/m^2^. The mean CD4+ T-cell count was 554 ± 259.68 cells/μL, viral load was ≤50 copies HIV RNA/mL plasma, the mean time patients had ≤50 copies HIV RNA/mL plasma was 76.02 ± 64.81 months and the mean CRP level was 5.57 ± 5.43 mg/L. The average HIV infection and total cART durations among patients were 92.42 ± 72.22 months and 82.02 ± 64.81 months, respectively. The mean RTL was 2.50 ± 1.87. None of the observed baseline characteristics were correlated with RTL. These results are presented in Table 3. Hyperlipidaemia had no influence on the RTL value of the patients (*p* = 0.94). The difference in the mean value of RTL in patients without hyperlipidaemia, patients with hypertriglyceridaemia, patients with hypercholesterolaemia, or mixed hyperlipidaemia (hypertriglyceridaemia and hypercholesterolaemia) was not significant. Regarding therapy for hyperlipidemia, there was no difference in the RTL outcome in patients who had not received therapy or who took statins or fibrates (*p* = 0.342). RTL did not differ significantly between patients with low, normal, or high blood glucose levels (*p* = 0.082). The presence of diabetes mellitus type 2 (in eight patients) had no significant effect on RTL (*p* = 0.6952). Only seven patients were taking insulin and their RTL did not differ from the RTL of patients who were not taking diabetes therapy (*p* = 0.2721). Regarding hypertension as a variable potentially associated with RTL, patients with high blood pressure did not have significantly different RTL compared to patients with normal blood pressure (*p* = 0.889).

The mode of HIV transmission did not affect RTL, patients infected by a homosexual partner, patients infected by a heterosexual partner, patients infected by intravenous drug use, vertical transmission, blood transfusion, and patients with an unknown mode of transmission did not have significantly different RTL lengths (*p* = 0.882).

**Figure 1:**
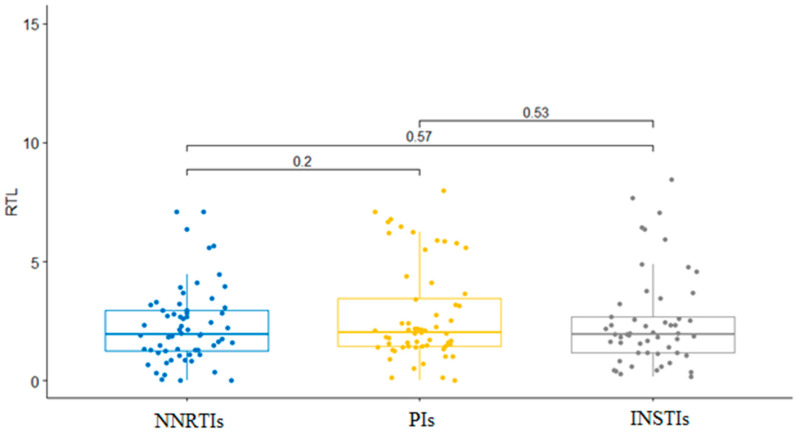
Boxplots of RTL values comparison between cART groups.

Of the176 patients, 53 (30.1%) had been using INSTIs for average of 18.86 ± 18.75 months, 60 (34.1%) had been using PIs for 23.60 ± 23.03 months, and 63 (35.8%) had been using NNRTIs for 37.38 ± 29.96 months, together with two NRTIs as a backbone. NRTIs combination, lamivudine with abacavir, was used by 111 patients (63.1%). In all, 60 patients (34.1%) were using tenofovir with emtricitabine and five (2.8%) patients were using lamivudine with zidovudine. As far as the INSTIs group is concerned, dolutegravir was used by more patients than raltegravir: 40 (75.5%) vs. 13 (24.5%), respectively. Within PIs, 48 patients (80%) used darunavir boosted with ritonavir. Nine (15%) patients were taking fosamprenavir with ritonavir, while only three (5%) patients were taking lopinavir together with ritonavir. Within the NNRTIs group, efavirenz was a drug used by 53 (84.1%) patients and nevirapine was used by 10 (15.9%) patients. The Kruskal–Wallis test showed no significant difference (*p* = 0.761) in RTL between the three therapy groups listed: RTL in patients taking INSTIs was 2.49 ± 1.89, RTL in patients taking PIs was 2.73 ± 2.03, and RTL in patients taking NNRTIs was 2.30 ± 1.62. The results of the Kruskal–Wallis test to determine the association between therapeutic groups and RTL are shown in Figure 1.

RTLs in different regimens within the cART groups are presented in Table 4. Kruskal–Wallis (for three groups) and Mann–Whitney (for two groups) tests showed no statistical significance between RTL among patients taking different drugs within the NRTIs group (*p* = 0.277), or INSTIs (*p* = 0.187), nor in the group of PIs (*p* = 0.790).

**Figure 2:**
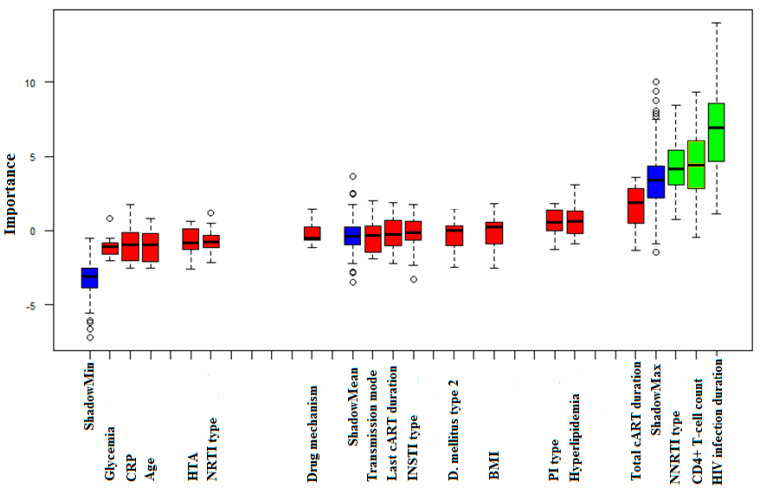
Boxplot of machine learning results, suggesting impact of different variables on RTL. ShadowMax, ShadowMean, and ShadowMin are Z-score values in duplicated and randomly shuffled data in the first steps of the ′Boruta′ algorithm used to determine variable significance in the dataset under study and are shown in blue. Variables identified as non-significant by machine learning are shown in red on the chart, while variables identified as significant are shown in green.

The results of the multiple regression analysis showed a slight negative effect of integrase inhibitors on RTL with a coefficient value of −1.13 and a *p*-value of 0.047. However, the overall model had low significance (F-statistic *p*-value = 0.7) and the bivariate association between RTL and integrase inhibitor type was not significant (*p*-value = 0.69). The statistical power of the multiple regression was 0.78 with an effect size of 0.1. The results of unsupervised machine learning rendered by the ‘randomForest’ and ‘Boruta’ R software packages suggested three variables with significant impact on RTL: duration of HIV infection, CD4+ T-cell count, and cART containing NNRTI. These results are visualized in Figure 2. However, consecutive Kendall’s correlation test found no significant association between duration of HIV infection and RTL (*p* = 0.220). Also, machine learning indicated the possible association of CD4+ T-cell count with RTL, but Kendall’s correlation test failed to confirm CD4+ T-cell count as a variable significantly associated with RTL (*p* = 0.536). Yet, the type of NNRTI patients were taking was a variable pointed to as significant by machine learning and the Mann–Whitney test showed that HIV-infected patients using efavirenz had significantly shorter telomeres (2.10 ± 1.55) when compared to patients taking nevirapine (3.33 ± 1.63) within cART (*p* = 0.018).

## 4. Discussion

The high demand for complex processing of large amounts of data, obtaining precise and reliable results and the ability to predict outcomes as accurately as possible is leading to the increased use of sophisticated data analysis techniques. The use of machine learning as an approach in pharmacogenetics helps identify patients at risk based on inter-individual variations in DNA sequences related to drug response, in line with the principles of precision medicine. In our study, machine learning indicated the potential impact of CD4+ T-cell count, duration of HIV infection, and cART containing NNRTI on telomere length in HIV-infected individuals. Similar to our findings, other authors such as Auld [50], Zanet [15], Blanco [18], and more recently Dragovic [20], found no association between telomere length and CD4+ T-cell count, time since HIV diagnosis, or cART type. In contrast to our results, few studies have shown that CD4+ T-cell count and the duration of HIV infection were significantly associated with telomere length [28,51,52,53]. Although older studies indicated a negative effect of cART on telomere length [24,51,54], several recent studies presented different results. Recent findings have shown that initiating suppressive cART has a positive effect on telomeres and reverses their decrease in length [24,55,56]. Not only that telomere length increases during suppressive cART, but also that T cells increase their replicative potential while telomeres become longer [51]. The importance of cART is also confirmed by the fact that cART interruption neutralizes its own beneficial effects on telomere length [57].

Despite the results of machine learning, our final analysis showed that only cART containing NNRTI had statistically a significant effect on telomere length: specifically, patients receiving efavirenz had significantly shorter telomeres than patients receiving nevirapine. To the best of our knowledge, to date, there are no published data on changes in telomere length in patients taking neither efavirenz nor nevirapine within cART. Although efavirenz and nevirapine are no longer preferred first-line regimen drugs in international guidelines treatment options, these medications are still widely used worldwide [58] and have the potential to make up a share of global prescribing practice. Efavirenz is known to have deleterious effects on cells by altering calcium homeostasis, decreasing creatine kinase activity, and increasing levels of proinflammatory cytokines [21], all leading to telomere shortening [19,59,60]. Efavirenz, used in therapeutic doses, binds to and modulates the opening and closing of the rianodine receptors [61] present in membranes of endoplasmic and sarcoplasmic reticulum in cells of almost all tissues [62,63]. Further on, cytoplasmic Ca^2+^ increases and Ca^2+^ stored within the endoplasmic reticulums is depleted, as much as five times faster than the basal rate [64]. Also, efavirenz significantly increases the presence of reactive oxygen species [20]. Efavirenz inhibits complex I of the mitochondrial electron transport chain, reducing mitochondrial oxygen consumption and membrane potential, leading to an increase in reactive oxygen species production [65]. In addition, efavirenz has been linked to the degradation of the protein p53 [22], which binds to fragile chromosome sites, such as telomeres, and enhances the ability of these regions to repair and replicate by directly stabilizing telomeres in response to DNA damage [23]. Since efavirenz prevents the intracellular survival of the protein involved in maintaining the length of telomeres, telomeres will inevitably shorten.

On the other hand, nevirapine did not affect p53 levels in cultured cells [66] but did significantly increase the expression of the transcription factor paired box 8 (PAX8) [67], known for its role in transactivating the promoters of the genes for the catalytic subunit of telomerase and the RNA component of telomerase, thereby upregulating telomerase activity [68].

Telomerase activity can differ in different blood cell types, and cell–type-specific differences in telomere length have been identified between six blood cell types, which are also differentially sensitive to ageing [69]. The rates of telomere shortening appear to differ not only between CD4 cells and CD8 T cells and B cells, but also within these cell lines, namely between naïve and memory cells [70]. To avoid losing some of the information by selecting individual cell lines, we chose to use mononuclear cells. The main reasons for using mononuclear cells in this study was their dynamic nature, their ability to represent the current physiological state of the body very well, and the proven fact that their telomere length generally reflects the state of telomere maintenance in other tissues [71,72].

The advantage of our study is that it was performed only on Caucasians, males, thus excluding the non-negligible influence of race and gender on telomere length [73]. The strength of our study is also reflected in the very strict inclusion criteria and exclusion criteria, aiming to eliminate the potential impact of all possible confounding factors on RTL. A potential weakness of this study is its relatively small sample size. In addition, we did not compare telomere length between HIV-infected patients and an HIV-uninfected matched cohort because our research was methodologically designed and our objectives set were such that we did not need a control group.

## 5. Conclusions

Among a variety of demographic and therapy variables, like the duration of HIV infection, total duration of cART, CD4+ T-cell count, or C-reactive protein (CRP) level, etc., we identified only cART with NNRTIs is impacting telomeres. More precisely, patients using efavirenz within cART had significantly shorter telomere length than patients using nevirapine within cART.

## Figures and Tables

**Table 1:** qPCR ingredients used for RTL determination and their volumes (per reaction).

**Reagent**	DNA	Forward primer (telomeres)	Reverse primer (telomeres)	Forward primer (HBG)	Reverse primer (HBG)	2 × Master Mix (Green/ ROX qPCR)	Water
**Quantity**	10 ng	1 μL	1 μL	1 μL	1 μL	12.5 μL	To a final volume of 25 μL

**Table 2:** The sequences, concentrations, and annealing temperature of the primers.

Primer	Sequence	Final Concentration	Annealing Temperature
TEL 1 forward	5′ CGGTTTGTTTGGGTTTGGGTTTGGGTTTGGGTTTGGGTT 3′	600 nM	56 °C
TEL 2 reverse	5′ GGCTTGCCTTACCCTTACCCTTACCCTTACCCTTACCCT 3′	600 nM	
HBG 1 forward	5′ TCTGACACAACTGTGTTCACTAGC 3′	300 nM	
HBG 2 reverse	5′ TCTGACACAACTGTGTTCACTAGC 3′	700 nM	

**Table 3:** Baseline patient characteristics and their correlation with RTL.

Variable	Mean Value ± SD	*p* Values of Kendall’s Correlation Test with RTL
Age (years)	42.70 ± 13.18	*p* = 0.904
BMI (kg/m^2^)	24.51 ± 3.49	*p =* 0.551
CD4+ T-cells count (cells/μL)	554 ± 259.68	*p =* 0.536
CRP (mg/L)	5.57 ± 5.43	*p* = 0.264
HIV infection duration (months)	92.42 ± 72.22	*p* = 0.220
Total cART duration (months)	82.02 ± 64.81	*p* = 0.095

**Table 4:** Telomere length in HIV patients within different cART groups.

cART Group	Regimen within the cART Group	Patients Receiving Regimen (%)	RTL (Mean Value ± SD)	Regimen’s Association with RTL
INSTIs	Dolutegravir	40 (75.5%)	2.69 ± 2.11	*p* = 0.187
	Raltegravir	13 (24.5%)	1.87 ± 1.37	
PIs	Darunavir + Ritonavir	48 (80%)	2.68 ± 1.99	*p* = 0.790
	Fosamprenavir + Ritonavir	9 (15%)	2.82 ± 2.34	
	Lopinavir + Ritonavir	3 (5%)	3.23 ± 2.33	
NNRTIs	Efavirenz	53 (84.1%)	2.10 ± 1.55	*p* = 0.018
	Nevirapine	10 (15.9%)	3.33 ± 1.63	
NRTIs	Lamivudine + Abacavir	111 (63.07%)	2.44 ± 1.84	*p* = 0.277
	Tenofovir + Emtricitabine	60 (34.09%)	2.00 ± 1.94	
	Lamivudine + Zidovudine	5 (2.84%)	3.59 ± 1.60	

## Data Availability

Data are available upon request to the authors.
